# Large-Area, Cost-Effective, Ultra-Broadband Perfect Absorber Utilizing Manganese in Metal-Insulator-Metal Structure

**DOI:** 10.1038/s41598-018-27397-y

**Published:** 2018-06-15

**Authors:** Majid Aalizadeh, Amin Khavasi, Bayram Butun, Ekmel Ozbay

**Affiliations:** 1 Department of Electrical and Electronics Engineering, Bilkent University, Ankara, 06800 Turkey; 2 Nanotechnology Research Center (NANOTAM), Bilkent University, Ankara, 06800 Turkey; 3 Electrical Engineering Department, Sharif University of Technology, Tehran, 11155-4363 Iran; 4 National Nanotechnology Research Center (UNAM), Bilkent University, Ankara, 06800 Turkey; 5 Department of Physics, Bilkent University, Ankara, 06800 Turkey

## Abstract

Achieving broadband absorption has been a topic of intensive research over the last decade. However, the costly and time consuming stage of lithography has always been a barrier for the large-area and mass production of absorbers. In this work, we designed, fabricated, and characterized a lithography-free, large-area compatible, omni-directional, ultra-broadband absorber that consists of the simplest geometrical configuration for absorbers: Metal-Insulator-Metal (MIM). We introduced and utilized Manganese (Mn) for the first time as a very promising metal for broadband absorption applications. We optimized the structure step-by-step and compared Mn against the other best candidates introduced so far in broadband absorption structures and showed the better performance of Mn compared to them. It also has the advantage of being cheaper compared to metals like gold that has been utilized in many patterned broadband absorbers. We also presented the circuit model of the structure. We experimentally achieved over 94 percent average absorption in the range of 400–900 nm (visible and above) and we obtained absorption as high as 99.6 percent at the wavelength of 626.4 nm. We also experimentally demonstrated that this structure retains broadband absorption for large angles up to 70 degrees.

## Introduction

Electromagnetic (EM) wave absorbers have attracted a lot of interest in technology, because of their vast range of applications in photovoltaics^1–4^, shielding^5,6^, thermal imaging^7–9^, sensing^10–12^, photodetection^13–17^, and thermal emission-based cooling^18,19^. With the advent of plasmonics^20^ and metamaterials, it became possible to design absorbers with ultra-thin sub-wavelength thicknesses, which is of high importance for optical packed circuit integration and the reduction of mass and cost of structures.

The general requirements for an absorber structure to absorb EM energy are having impedance matching with free space to minimize reflection^21,22^, as well as near to zero transmission. One of methods to absorb light is to trap EM wave in a resonance mode of light and to absorb it by means of loss mechanisms such as electron collisions. The common resonance modes used in the design of absorbers are Localized Surface Plasmon (LSP) modes^23–27^, Propagating Surface Plasmon (PSP) modes^28,29^ and Fabry-Perot resonance modes^30,31^. To obtain broadband absorption, we can broaden the resonance bandwidth by sufficiently lowering the quality-factor with the appropriate choice of metals and the geometric design. The other approach is to design the structure in a way to have the superposition of several adjacent resonance modes and, therefore, broaden the absorption spectrum.

It is of high importance for the broadband absorber to be designed in a way to have large-scale production compatibility. One big barrier to large-scale production is the use of lithography in the structures, which is time consuming and costly. In order to have mass production compatibility, it is also very beneficial to use inexpensive materials in the structure. Therefore, designing a broadband lithography-free absorber in which inexpensive materials are used is very advantageous and meets the requirements of large-scale production.

In one of the pioneer works that was based on the superposition of LSPs, Aydin *et al*.^32^ achieved an average of 71 percent absorption in the visible region (400–700 nm), by superposing the LSP modes of varying width of nanorods. However, it was achieved by use of Electron Beam Lithography (EBL). Some other works are fabricated or designed to obtain broadband absorption in the visible^33–35^, near-infrared (NIR)^36,37^, mid-infrared (MIR)^38–41^, and Terahertz^42–45^ ranges by using EBL and implementing the above-mentioned ideas.

In a recent lithography-free work conducted in our group, 97 percent absorption was achieved from 400 to 2000 nm wavelength by employing the superposition of LSP modes of dielectric-metal core-shell nanowires. This broadband perfect absorption was the result of randomness in the size of chemically synthesized nanowires^46^. One other common method to broaden the absorption band without the use of lithography is to anneal the structure in high temperatures, which is called the De-wetting process^47^. This leads thin layers to reform into nanoholes or nanoparticles, and adds the superposition of the LSP modes of nanopatterns into the absorption bandwidth. Some works are done in our group to broaden the absorption of Metal-Insulator-Metal-Insulator (MIMI) structure using this method^48,49^. However, this comes with the cost of access to very high temperatures, which is time consuming and always brings the possibility of sample cracking, especially for the ones with thin substrates.

As mentioned earlier, lowering the quality factor of Fabry-Perot resonance is another way to achieve broadband absorption without lithography. This resonance can be obtained by making a Fabry-Perot resonator using an MIM structure. By adding the number of Metal-Insulator (MI) pairs, a broader bandwidth can be obtained. Some lithography-free works have achieved broadband absorption by employing MI multi-layer stacks. These works are done by using different combinations of MI pairs such as Cr-SiO_2_^50^, W-Al_2_O_3_^51^, Ti-SiO_2_^52^, and W-Al_2_O_3_-Ti- Al_2_O_3_^53^. Also in a recent lithography-free work, broadband infrared absorption is obtained by implementing ITO-photoresist pairs^54^. These MI-stack absorbers have at least four layers (two MI pairs) in their structure. However, the purpose of our work is not to design an MI-stack absorber. We want to design a broadband absorber using the tri-layer MIM structure, which is the simplest possible configuration for absorbers. Moreover, as mentioned before, the MI-stack absorbers are discussed in several works, but, to the best of our knowledge, there are very few tri-layer broadband, lithography-free and annealing-free absorbers, reported previously^55^.

In the present paper, we experimentally demonstrate lithography-free, ultra-broadband nearly perfect absorption within 400–900 nm range by the MIM structure. The measured average absorption in this wavelength range is above 94 percent by the use of Mn in the metal layers. The most important difference between our work and earlier works is that we introduce Mn for the first time in broadband absorbers, and, by detailed analysis, show that it is a very promising metal for achieving maximum absorption in broadband absorption applications. It is also an inexpensive metal unlike gold or silver, which can reduce the cost of fabrication in favor of mass production. Also, the proposed structure does not need post-annealing.

In the following parts of the paper, first, MIM cavities and formulation of their resonance modes is discussed. Then, the optimization process of this structure is explained in detail in terms of two factors: 1. Materials, and, 2. Dimensions. First, to optimize the structure by choosing the appropriate materials, the effect of dielectric material is investigated, and also it has been demonstrated numerically that the best choice of metal is Mn for this structure. During the optimizations and calculations, the structure is modeled by using the Transfer Matrix Method (TMM) and also circuit theory. Some of simulations are also performed by the Finite Difference Time Domain (FDTD) method. It is also conceptually demonstrated as to how Mn is such an appropriate metal for the MIM structure and for broadband absorbers in general, by discussing based on its permittivity profile. Then, the structure is optimized in terms of the dimensions of different layers. At the end, the optimal structure has been fabricated and characterized. The experimental results are in good agreement with simulations and calculations showing a very large absorption bandwidth.

## Calculation and Analysis

**Figure 1:**
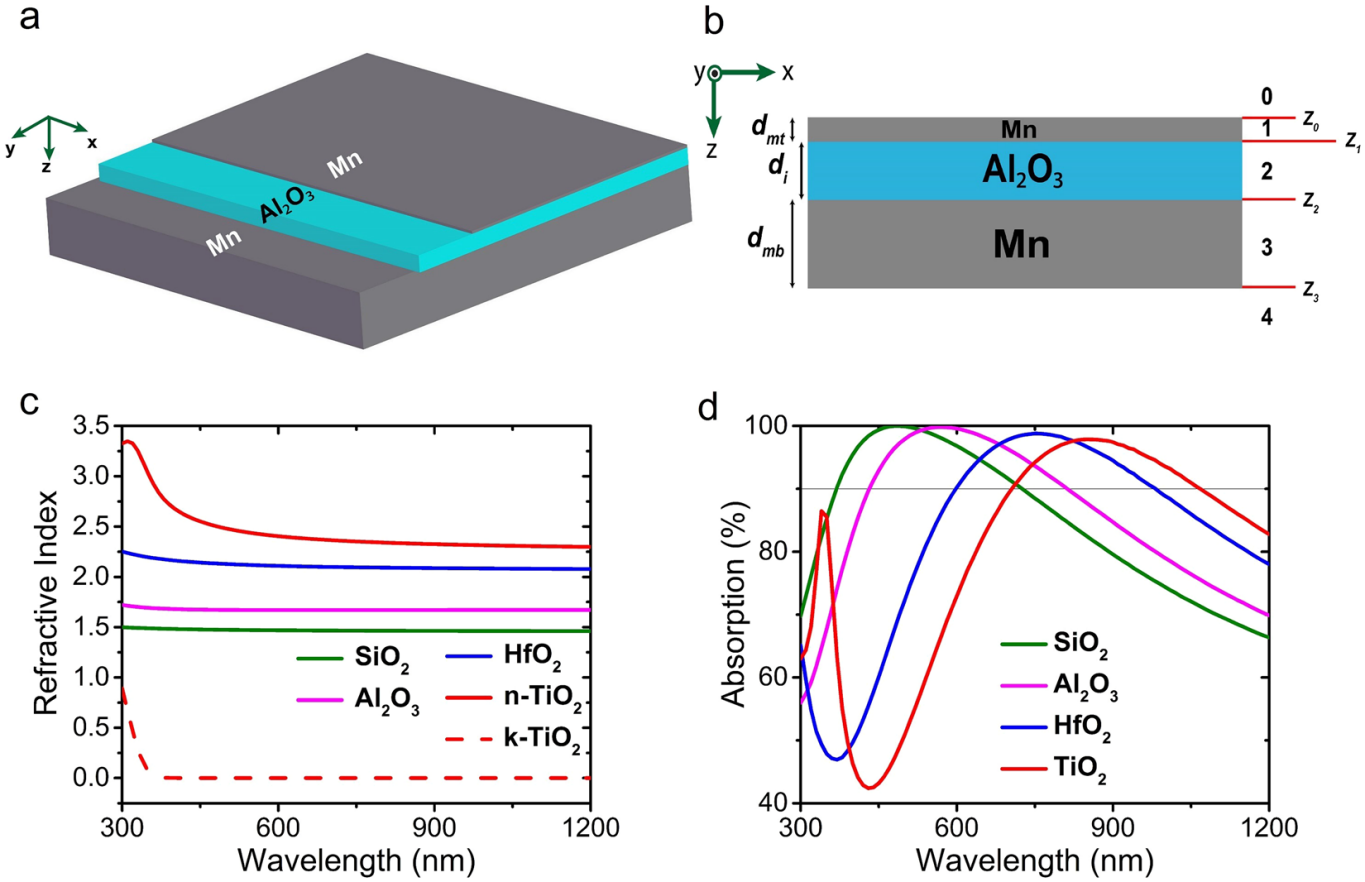
(**a**) 3D schematic of the proposed MIM structure, (**b**) cross section of the structure in xz plane, (**c**) refractive index of four common dielectrics investigated in the absorption calculations and, (**d**) calculated absorption spectrum of MIM structure with dielectric layer chosen as those plotted in (c), the metal layers assumed as Mn and ${d}_{i}$ = 65 nm.

Figure 1(a) depicts a 3D graphical schematic of the MIM structure and Fig. 1(b) shows its 2D cross section in xz plane. The bottom layer is an optically thick metal layer that is much thicker than the skin depth of the metal to ensure zero transmission. The middle insulator layer can be chosen among different dielectrics, taking into account the fact that the refractive index of the dielectric is an important parameter in determining the resonance frequency of the cavity mode. The top layer must be optically thin to allow the coupling of the incident light into the MIM cavity and consequently getting trapped inside it with back and forth reflections from metals. The trapped light is partly absorbed in each reflection, eventually, leading to complete absorption.

The resonance mode of the asymmetric Fabry-Perot cavity shaped by this MIM structure follows the equation of the form^30^

1 $$2(\frac{2\pi }{{\lambda }_{res}}){n}_{i}{d}_{i}+{\varphi }_{b}+{\varphi }_{t}=2\pi m$$

where *λ*_*res*_ is the resonance wavelength, *n*_*i*_ and *d*_*i*_ are the refractive index and thickness of the insulator layer, respectively, and $m$ is an integer number which determines the order of cavity mode. It can be deduced from equation (1) that higher order cavity modes resonate at smaller wavelengths. *φ*_*b*_ and *φ*_*t*_ are the phase shift acquired from reflection from the bottom and top metal layers, respectively. It is noteworthy that equation (1) along with all other analytical theories presented throughout the paper is only applicable for normal incidence. It can be seen that by increasing *n*_*i*_ or *d*_*i*_, *λ*_*res*_ increases as well. In other words, by increasing the optical beam path which is written as *n*_*i*_*d*_*i*_, the resonance wavelength red-shifts. Therefore, to modify the center of the absorption band, one can use dielectrics with different refractive indices or adjust its thickness according to the absorption band of desire. To achieve broadband absorption, the quality-factor of cavity mode should be sufficiently low, which is essentially obtained by the correct choice of metal.

To calculate absorption (A), reflection (R) and transmission (T) must be calculated and then absorption can be obtained from the A = 1 − R − T formula. In the present paper, TMM is used for these calculations, and the results are verified by FDTD simulations and circuit theory calculations. It should be noted that, for a sufficiently thick bottom metal layer, T is almost zero and, therefore, the absorption is simplified to A = 1 − R. To be more precise, however, we include T in our calculations.

## Material Optimizations

In this part, the effect of dielectric material and the metal is investigated and the optimized materials are chosen among them.

Figure 1(c) shows the refractive index of four different common dielectrics: SiO_2_, Al_2_O_3_, HfO_2_ and TiO_2_, and Fig. 1(d) shows the calculated absorption spectrum of MIM using the four mentioned dielectrics, while keeping the metal (Mn) and all dimensions constant. The thickness of the layers from bottom to top is 200, 65, and 5 nm, respectively. As expected, the absorption mode red-shifts as the refractive index of the dielectric material increases. Since we have the possibility to deposit Al_2_O_3_ by Atomic Layer Deposition (ALD) which deposits layers with lower roughness, the dielectric material is chosen to be Al_2_O_3_ for the rest of this work. It has to be mentioned that based on previous explanations and as it will be demonstrated in detail in the following sections, this thickness of the insulator layer, 65 nm, has been chosen by using Al_2_O_3_ to obtain high absorption in the desired bandwidth of 400–900 nm. It should be noted that other dielectrics can also be used, considering the fact that in order to achieve high absorption in the same wavelength band, the thickness of the dielectric layer should be calculated accordingly and the optimized thickness for this layer will be different from 65 nm.

In order to choose the most appropriate metal, as it will be elevated in detail in the next parts, the input impedance of the structure should be calculated. Therefore, first the calculation of the input impedance is explained. The input impedance of the proposed three-layer structure is calculated by circuit theory.

**Figure 2:**
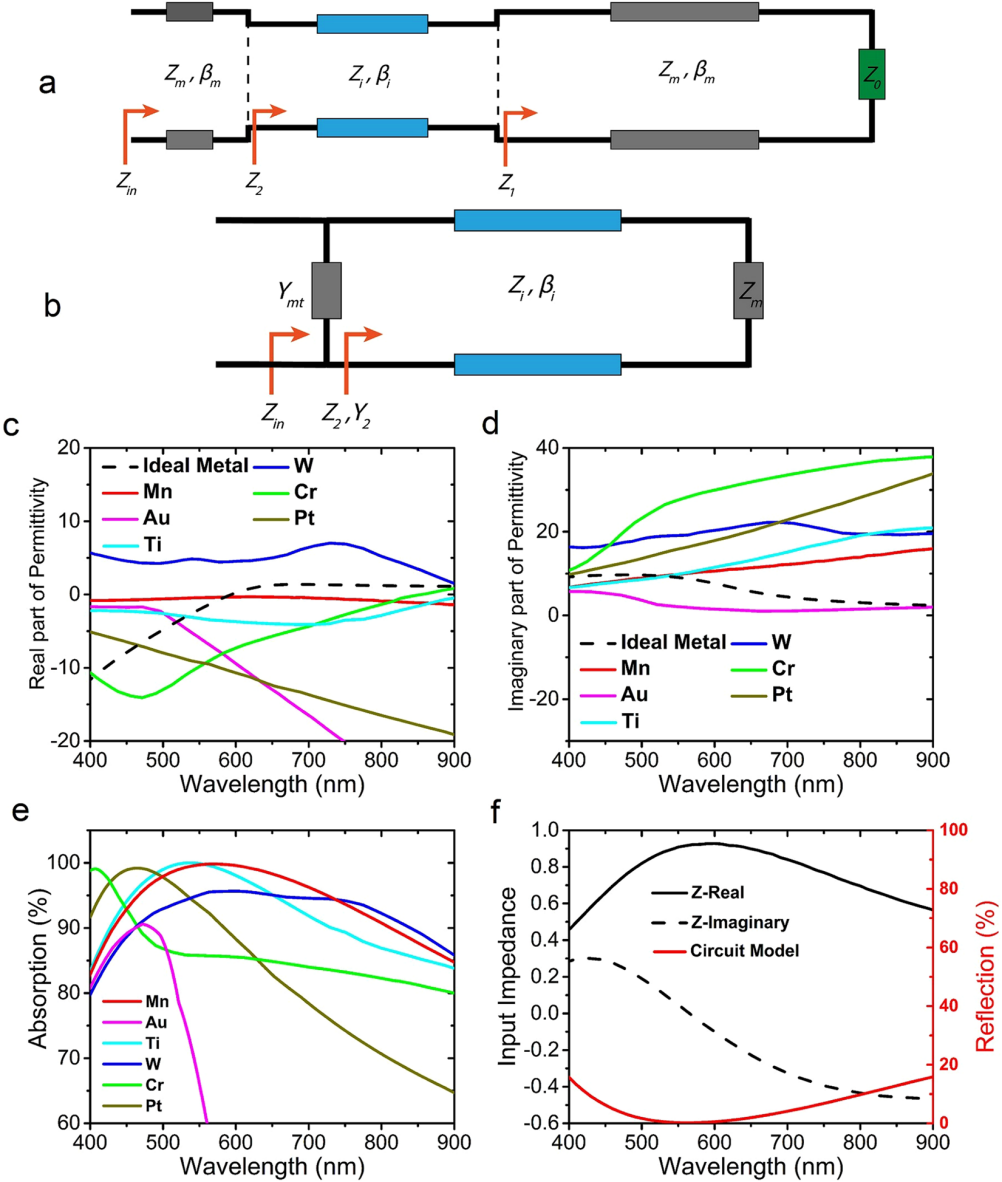
(**a**) Equivalent circuit of the MIM structure, (**b**) simplified equivalent circuit model of the MIM structure, (**c**) real and (**d**) imaginary part of permittivity of Mn and the ideal metal, along with those of Ti, W, Cr, Pt, and Au that are commonly reported metals for broadband absorption, (**e**) calculated absorption spectrum of the MIM structure using metals mentioned in (c) and (d). (**f**) Real and imaginary part of input impedance of the structure using Mn (black solid and dashed lines), normalized to free-space impedance, and reflection calculated using circuit theory (red line).

The equivalent circuit of the structure is shown in Fig. 2(a). At normal incidence, *β*_*m*_ and ${Z}_{m}$ are the propagation constant and characteristic impedance (normalized to the impedance of free space) of the transmission line corresponding to the metal layer and are equal to *n*_*m*_*β*_0_ and $1/{n}_{m}$, respectively, with *n*_*m*_ denoting the refractive index of the metal and ${\beta }_{0}$ denoting the propagation constant of light in vacuum. *β*_*i*_ and *Z*_*i*_ are similarly the corresponding parameters for the transmission line modeling the insulator layer. Finally, *Z*_0_ is the normalized impedance of free space, which is equal to 1. It is assumed in this model that both the top and bottom metal layers have the same material, therefore, for both layers, the characteristic parameters are denoted with the subscript of ‘*m*’.

To better understand the mechanism of the absorption, we simplify the equivalent circuit by modeling the top thin metal layer with a surface conductivity, which is an accurate approximation for ultra-thin layers^39^. The characteristic equivalent admittance (surface conductivity) of the top layer (normalized to free space admittance) is

2 $${Y}_{mt}=j(1-{\varepsilon }_{m})\omega {d}_{mt}/c$$

where $c$ is the free space speed of light and $\omega$ is the angular frequency. *ε*_*m*_ denotes the relative permittivity of metal, and *d*_*mt*_ represents the top metal layer thickness. The above equation is obtained by approximating the bulk conductivity by surface conductivity when the layer is ultra-thin. Then the value of equivalent admittance of that layer is equal to the value of surface conductivity.

The second approximation is assuming the bottom metal layer thickness to be infinite. Therefore, *Z*_1_ in Fig. 2(a) can be replaced by a load impedance of *Z*_*m*_. Thus, the circuit model of the structure gets simplified into Fig. 2(b).

The input impedance seen from the interface of the top metal and the dielectric layer which is denoted as *Z*_2_ in Fig. 2(a,b) can be written as^56^

3 $${Z}_{2}={Z}_{i}\frac{{Z}_{m}-j{Z}_{i}\,\tan ({\beta }_{i}{d}_{i})}{{Z}_{i}-j{Z}_{m}\,\tan ({\beta }_{i}{d}_{i})}$$

In this approach, the admittance of top Mn layer or *Y*_*mt*_ is parallel to the equivalent input admittance of the other two layers which is equal to the inverse of input impedance of these two layers or *Z*_2_. The total input admittance then, is the sum of admittances *Y*_*mt*_ and *Y*_2_.

After calculating the input impedance (admittance) of the structure, the reflection of the structure, can be calculated by the following formula

4 $${\rm{R}}={(|\frac{{Z}_{in}-1}{{Z}_{in}+1}|)}^{2}$$

which is the well-known relation for reflection at the interface of two media with different impedances. Now, we approximate T to be zero, which is a very good approximation for a sufficiently thick bottom layer. We show the accuracy of this approximation by calculating transmission in the next sections. Therefore, the absorption can be simply calculated as A = 1 − R.

Now, for choosing the appropriate metal, we assume that the top and bottom metal layers both are chosen as the same material. Then we try to find the best metal for the structure. The material of metal layers plays the main role in determining the quality factor of the cavity. For this purpose, we calculate the normalized input impedance of the structure using circuit theory and then we equate it to the impedance of free space (i.e. 1). This is the condition for having zero reflection. From there the permittivity of the ideal metal can be calculated. Thereafter, the metal with the closest permittivity profile to the ideal metal should be chosen. During this calculation, the dimensions are 5, 65, and 200 nm, respectively and Al_2_O_3_ has been used as the insulator layer.

The calculated real and imaginary parts of relative permittivity profile of ideal metal as well as some other metals are shown in Fig. 2(c) and Fig. 2(d), respectively. The metals are Titanium (Ti), Tungsten (W), Platinum (Pt), Chromium (Cr), and Gold (Au). Apart from Au, other metals are the candidates reported so far for broadband absorption applications, especially for lithography-free structures. Au can be used for broadband absorption in the case of patterning. The permittivities of above-mentioned metals are plotted in these figures along with Mn, for a fine comparison between their permittivity profile and the one for ideal metal. The permittivity of the ideal metal is shown by black dashed lines in Fig. 2(c,d). As it is obvious from both Fig. 2(c,d), Mn has the closest permittivity profile to the ideal metal. It can be observed that the imaginary part of permittivity of the ideal metal is very close to that of Mn, from 400 nm up to around 600 nm. On the other hand, after 600 nm, up to the end of our desired spectrum, i.e., 900 nm, the real part of permittivity of Mn is similar to the real part of permittivity of the ideal metal. This demonstrates that Mn is the best candidate for our structure in our bandwidth of interest. So we choose Mn for the rest of our work as the top and bottom layer metal. It can be observed that around the calculated absorption peak wavelength, both the real and imaginary parts of permittivity of Mn almost intersect with the one for ideal metal. The material data of Mn and Al_2_O_3_ shown here, and also used in all the simulations and calculations have been obtained by fitting spectroscopic data obtained from ellipsometry measurements. Material data for all other metals except for Au are obtained from Palik^57^ and for Au the data are obtained from Johnson and Christy^58^.

Figure 2(e) shows calculated absorption when the 5 different mentioned metals are used, along with the case of using Mn. It is obviously notable that as predicted, Mn has the best absorption in terms of absorption strength and bandwidth at the same time. This is due to the fact that using Mn provides a better and broader impedance matching to free-space that leads to the minimization of reflection and trapping of light in a broad spectrum. Figure 2(f) shows the real and imaginary parts of the calculated input impedance of the structure normalized to that of free-space, as well as reflection calculated using equation (4). It can be seen from this figure that the reason of having a broad dip in the reflection spectrum (broad absorption peak) of this structure lies in the fact that the real part of input impedance is close to 1 in a broad wavelength band, while the imaginary part of it is close to zero. This minimizes the reflection and provides a broadband impedance matching to the free space.

**Figure 3:**
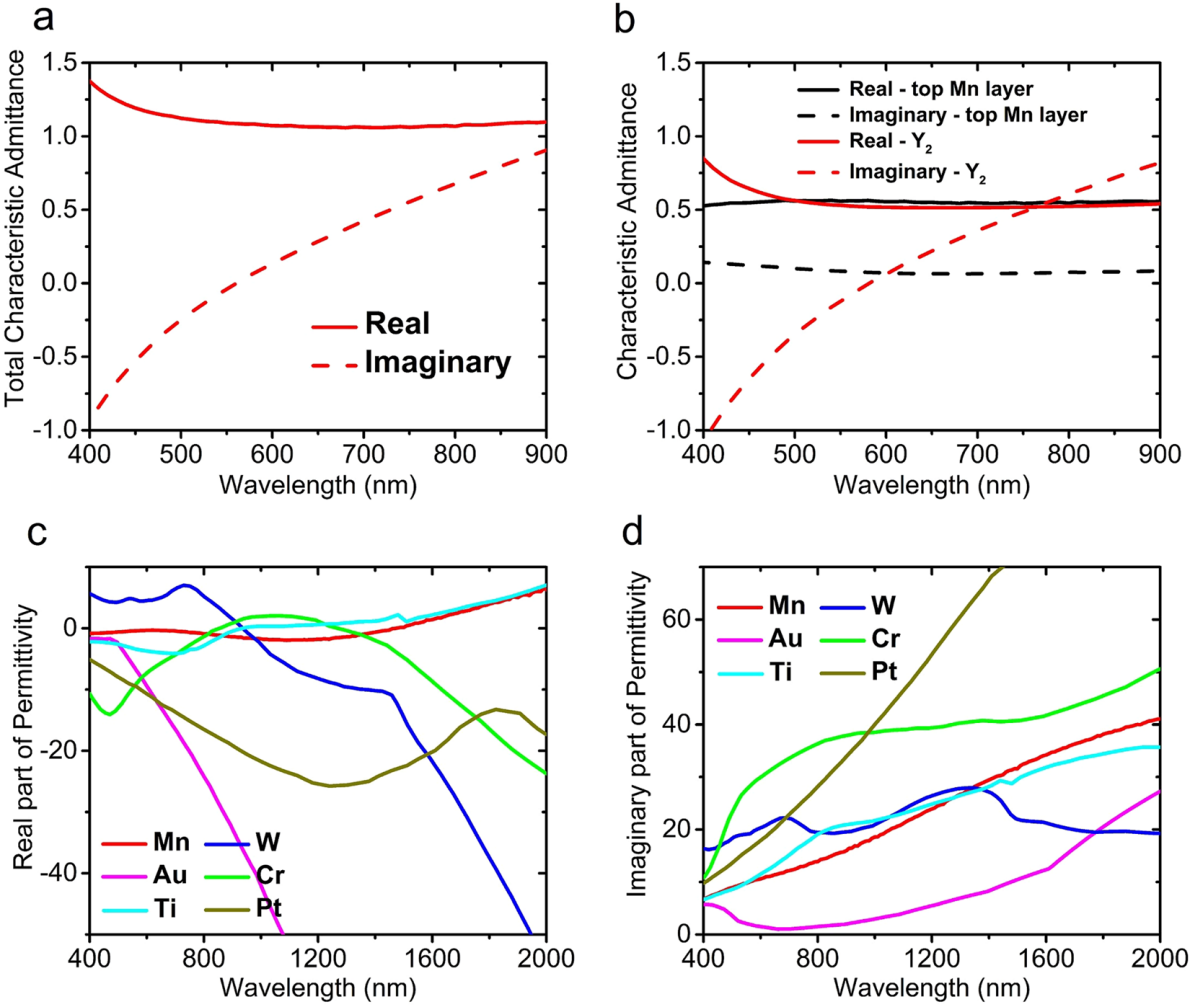
(**a**) Total characteristic admittance of the structure, calculated by circuit theory, (**b**) characteristic admittance of top Mn layer modelled as surface conductivity and characteristic input admittance of other two layers looking from top of Al_2_O_3_ (denoted as ${Y}_{2}$ in Fig. 2(b)), (**c**) real and (**d**) imaginary part of permittivity of Mn along with those of the other 5 metals, extended up to 2000 nm wavelength.

Figure 3(a) shows the input admittance of the structure. To have admittance matching with free space, similar to impedance matching, the real part of input admittance must be close to 1 and its imaginary part must be close to zero, which is the case here as it can be seen from Fig. 3(a). The admittance matching process reveals better why Mn is such an appropriate metal for this structure. The real and imaginary parts of the equivalent admittance of top Mn layer and the input admittance seen from the dielectric and the top metal interface which is denoted as ${Y}_{2}$ in Fig. 2(b), are shown in Fig. 3(b). It is obvious that the real part of *Y*_2_ is around 0.5 within the wavelength range of interest. The other required 0.5 of admittance for admittance matching is provided with the admittance of top Mn layer, as it can be seen in the figure. Figure 3(a) shows that the real part of input admittance of the structure stays around 1, but its imaginary part does not have such a behavior and its absolute value grows by getting far from the resonance wavelength. This limits near-to-perfect absorption to around 400–900 nm. It can be seen from Fig. 3(b) that the imaginary part of admittance of the top Mn layer is around zero and, therefore, has almost no contribution to admittance mismatch happening outside the bandwidth.

Figure 3(c,d) show the real and imaginary part of relative permittivity of Mn along with previously mentioned other 5 metals in a broad wavelength range between 400 and 2000 nm. By taking a look at these figures one can understand more about the physical reason behind broadband absorptive behavior of Mn. Compared to other metals, Mn has a very slowly changing real part of permittivity in the broad range of 400–2000 nm, covering visible and NIR, which makes impedance matching of the structure to that of free space easy. Its real part of permittivity has small values in this broad spectrum, which leads to high field penetration; and, at the same time it has a large imaginary part of permittivity that leads to high absorption. The lossy behavior of Mn results in a low quality factor Fabry-Perot MIM cavity. On the other hand, some other metals such as Gold and Silver have a very narrowband MIM cavity mode, because they are low-loss metals.

So far, we have thoroughly investigated the effect of materials and highlighted the importance of the choice of metals. The above discussions show what a great potential Mn has to be employed in broadband absorption structures, even in higher wavelength ranges. Unlike its high potential, it has not been employed in absorbers so far. Therefore, we choose Mn for metal layers and Al_2_O_3_ as the dielectric layer for the structure, and proceed to the optimization of dimensions in the following section.

## Dimensions Optimization

**Figure 4:**
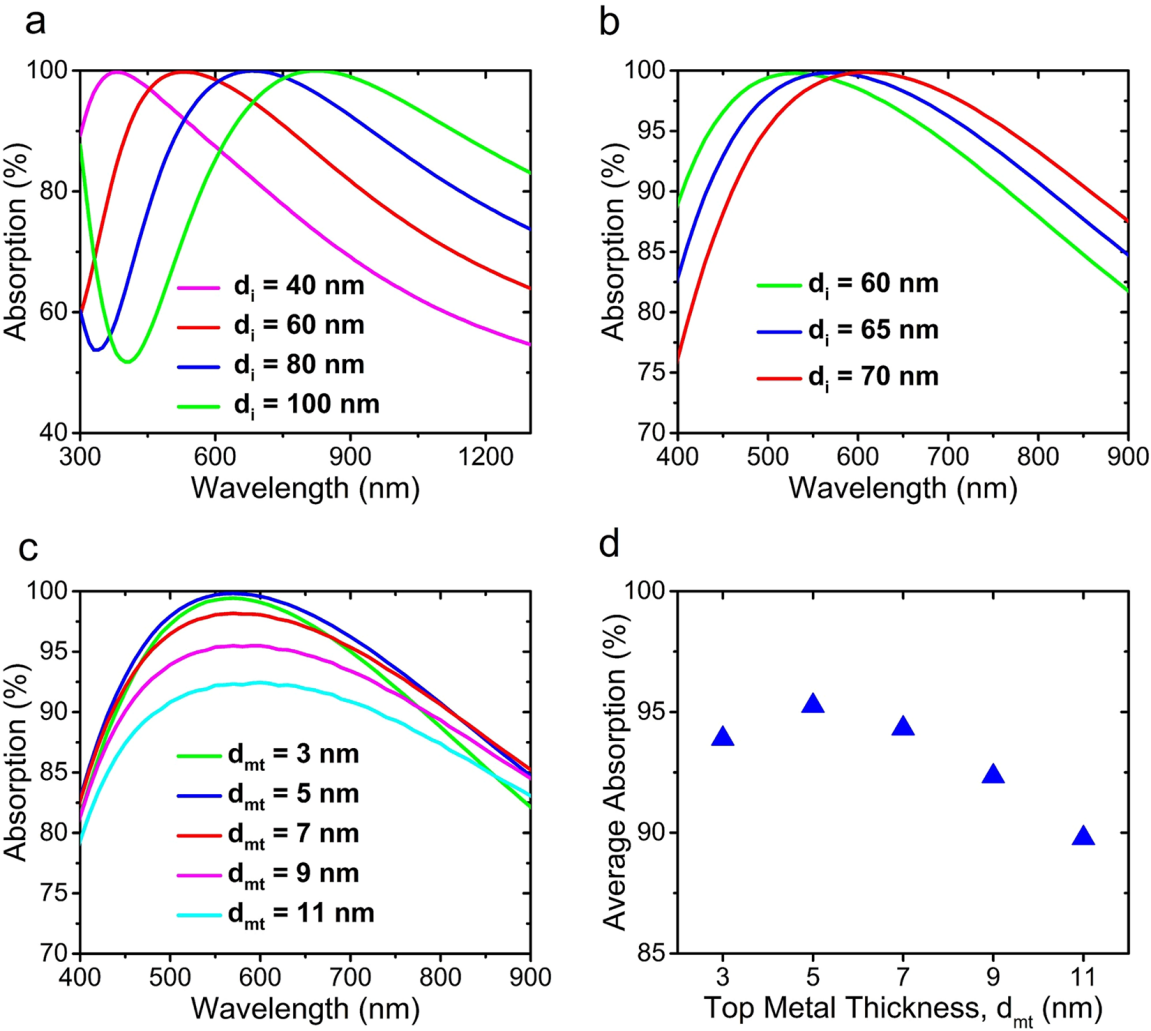
Calculated absorption spectrum of MIM structure for the different values of ${d}_{i}$ with (**a**) 20 nm steps and, (**b**) 5 nm steps, while Mn is assumed as the metal and the top layer thickness is ${d}_{mt}$ = 5 nm, (**c**) calculated absorption spectrum of MIM structure for different values of ${d}_{mt}$ and, (**d**) average absorption for different values of ${d}_{mt}$ calculated in the 400–900 nm wavelength range, again Mn is assumed as the metal and the thickness of the dielectric layer is ${d}_{i}$ = 65 nm.

As explained in the previous parts of the paper, by increasing the thickness of the insulator layer, the optical path of the light inside the structure increases and the absorption band experiences a red-shift (see equation 1). One way to tailor the optical beam path (*n*_*i*_*d*_*i*_) is changing the dielectric material that indeed changes *n*_*i*_ in equation 1 that was explained in the previous section. The other method is the control of *d*_*i*_. Figure 4(a) demonstrates the effect of the thickness of the insulator layer on the absorption spectrum. It can be observed that by choosing thicker dielectrics, the absorption band red-shifts. Among the calculated absorptions for different dielectric thicknesses, the one for 60 nm matches our desired spectrum, i.e. the visible range and some parts of NIR. For finely tuning the dielectric thickness, absorption spectrum has been calculated for different *d*_*i*_ values of 60, 65, and 70 nm as shown in Fig. 4(b). As it can be seen from Fig. 4(b), in the case of choosing 60 nm, the absorption strength will decrease in NIR range and, on the other hand, by choosing 70 nm as the dielectric thickness, absorption strength decreases in shorter wavelengths of visible range; but in the case of 65 nm, the best overall absorption strength and almost perfect absorption is obtained within the broad range of 400 nm to 900 nm wavelength. Therefore, *d*_*i*_ is chosen to be 65 nm.

Now, to choose the best thickness for the top metal layer or *d*_*mt*_, absorption for different values of *d*_*mt*_ ranging from 3 to 11 nm has been calculated and is shown in Fig. 4(c). It is obvious that the 5 nm is the best thickness for the top layer because it has the strongest and broadest response compared to the other *d*_*mt*_ values. Figure 4(d) illustrates the average absorption calculated in the 400 to 900 nm wavelength range versus *d*_*mt*_, again demonstrating that 5 nm is the best choice for the top layer thickness.

**Figure 5:**
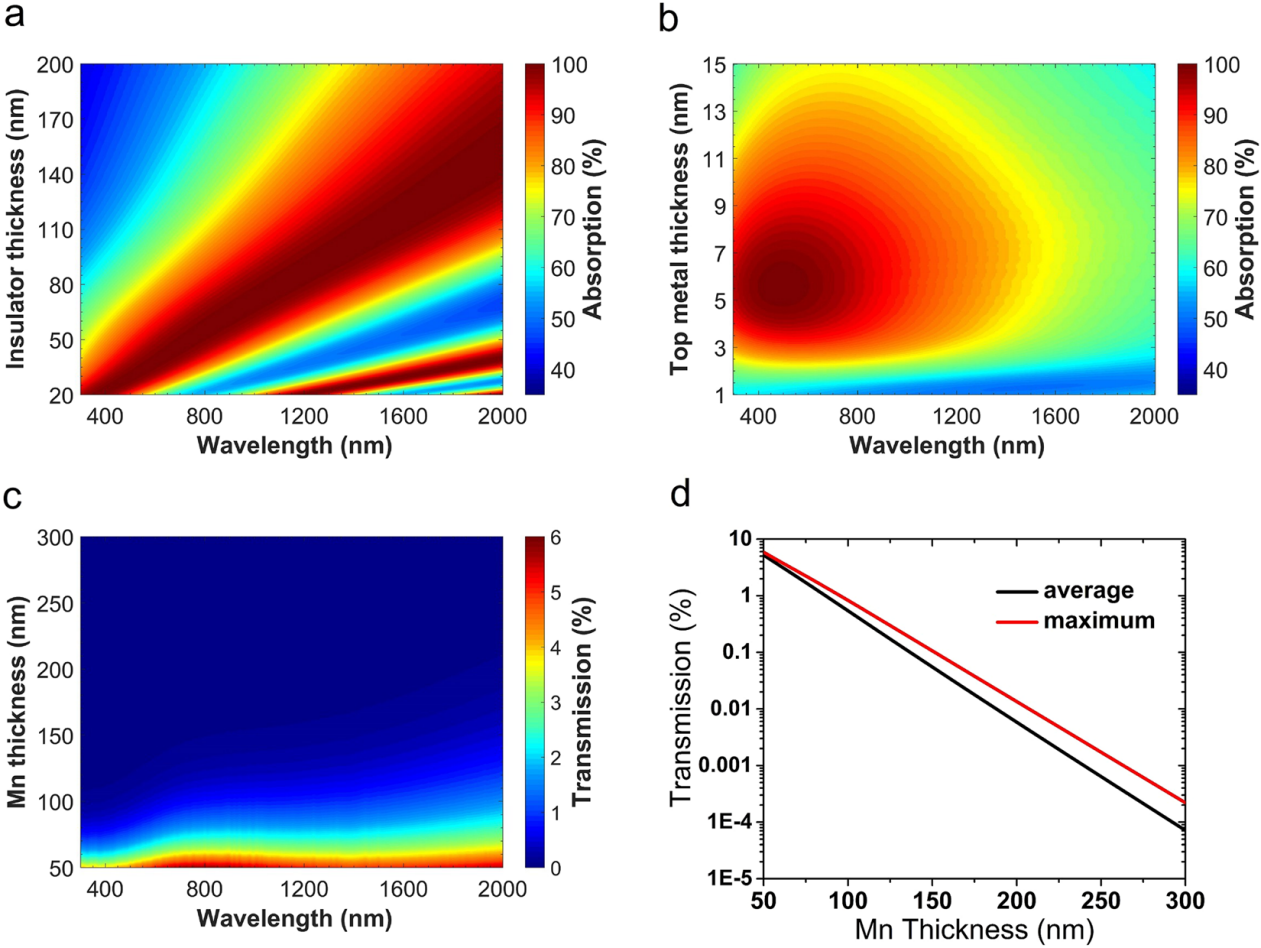
(**a**) Calculated absorption of MIM structure versus the wavelength and different values of ${d}_{i}$, assuming Mn as the metal with ${d}_{mt}$ = 5 nm and Al_2_O_3_ as the dielectric, (**b**) same as (**a**) but for different values of ${d}_{mt}$ and with ${d}_{i}$ = 65 nm, (**c**) calculated transmission from a layer of Mn versus wavelength and different thicknesses of Mn layer and, (**d**) maximum and average values of transmission from a layer of Mn versus Mn thickness, calculated in the 400–900 nm wavelength range.

To investigate the fabrication tolerance of the structure, which is a very important factor for mass production purposes, we have calculated the absorption of the structure for varying *d*_*i*_ versus wavelength and also for varying *d*_*mt*_ versus wavelength as shown in Fig. 5(a,b), respectively. These color-plots show that deviations from optimal dimensions in the order of a few nanometers do not affect the absorption bandwidth and strength, significantly.

As for the bottom layer, there is no optimization needed and it is only necessary to be thick enough so that it almost completely blocks the transmission. Figure 5(c) depicts the transmission for different thicknesses and varying wavelength and gives a good insight about this issue. There is a slight increase in transmission toward higher wavelengths that is due to the decrease of the optical path inside the material which leads to reduction of absorption of the light passing through it. Figure 5(d) shows the maximum and average transmission in the range of 400–900 nm versus Mn thickness. It is obvious that maximum transmission remains below 1 percent for thicknesses down to around 100 nm. Based on these figures, we conservatively set the thickness of the bottom Mn layer to 200 nm.

To summarize, the optimized structure is as follows: The dielectric is chosen to be Al_2_O_3_ and the metal is Mn. The dimensions of 5, 65 and 200 nm are chosen for the top metallic layer, dielectric insulator, and the bottom thick metal layer, respectively. In the next section, we present the experimental results for this structure.

## Results and Discussion

**Figure 6:**
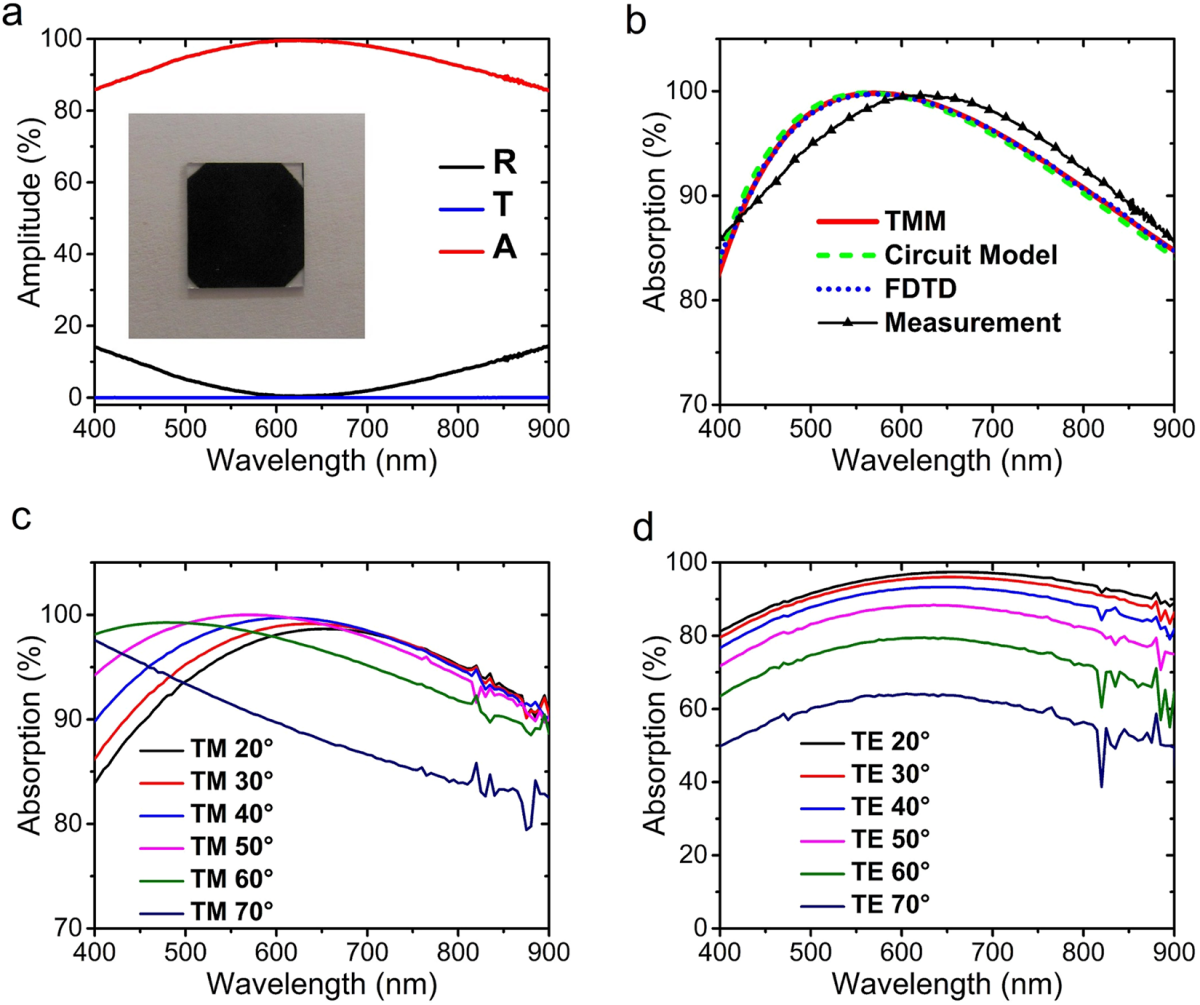
(**a**) Measured reflection, transmission and absorption spectrum of the fabricated MIM structure, inset: image of the fabricated sample, (**b**) comparison of simulation and calculation methods with measurement for the absorption spectrum of the optimal MIM structure, and, measured absorption of the MIM structure with different angles of incidence ranging from 20 to 70 degrees, for (**c**) TM and (**d**) TE polarizations of incident light. Note that y-axis range is different between (**c**) and (**d**).

The structure with optimized dimensions was fabricated and characterized. In order to avoid any contribution from the substrate to the absorption, and also to be able to measure the transmission of MIM, the structure has been fabricated on a transparent Sapphire substrate. Figure 6(a) shows the measured transmission, reflection and absorption of the structure for the case of normal incidence. The experimental result for absorption demonstrates over 94 percent average absorption in the broad range of 400–900 nm and has absorption as high as 99.6 percent at the wavelength of 626.4 nm. The image of the fabricated absorber is shown in the inset of Fig. 6(a) which demonstrates the black color of the absorber due to the strong absorption of the visible light. Moreover, as expected, the measurements confirm that the structure has zero transmission.

The experimental results are compared against simulation results calculated with the circuit model, TMM and FDTD, in Fig. 6(b). It is obvious that the simulation and experimental results are in good agreement. The insignificant mismatch between the simulation and experimental results may be due to using different deposition rates for the top and bottom metal layers (see Methods, Experimental). The permittivity profile of Mn has been extracted from a film deposited by the same rate as the bottom metal. Therefore, since using different deposition rates may affect the grain size which may consequently affect the optical properties, the optical properties of the top metal layer may be slightly different. However, this possible difference in the optical properties must have been insignificant because of the negligible mismatch between theory and experiment. Other possible causes may be variations in the thickness of the layers and also imperfections in the roughness of the top layer since it is very thin and it is hard to obtain ultra-thin metallic layers with no roughness while using physical vapor deposition methods. However, for broadband absorbers, roughness is a positive characteristic and contributes to the strength and also the broadening of the absorption because it leads to having the superposition of various modes in the absorption.

Now let us investigate the performance of the fabricated structure for oblique incidence and to show that it has an omnidirectional performance, which is very important in different applications. We measured the absorption of the structure for both TM and TE polarizations and for angles ranging from 20 to 70 degrees. Figure 6(c,d) show the absorption of the structure for the mentioned incident angles in the wavelength range of 400 to 900 nm, for TM and TE polarizations, respectively. It is obvious that the device retains its high and broadband absorption performance up to very large incident angles such as 70 degrees for TM polarization. However, the TE polarization is more sensitive to the variations of incident angle. Note that the range of y-axis is different for Fig. 6(c,d).

**Figure 7:**
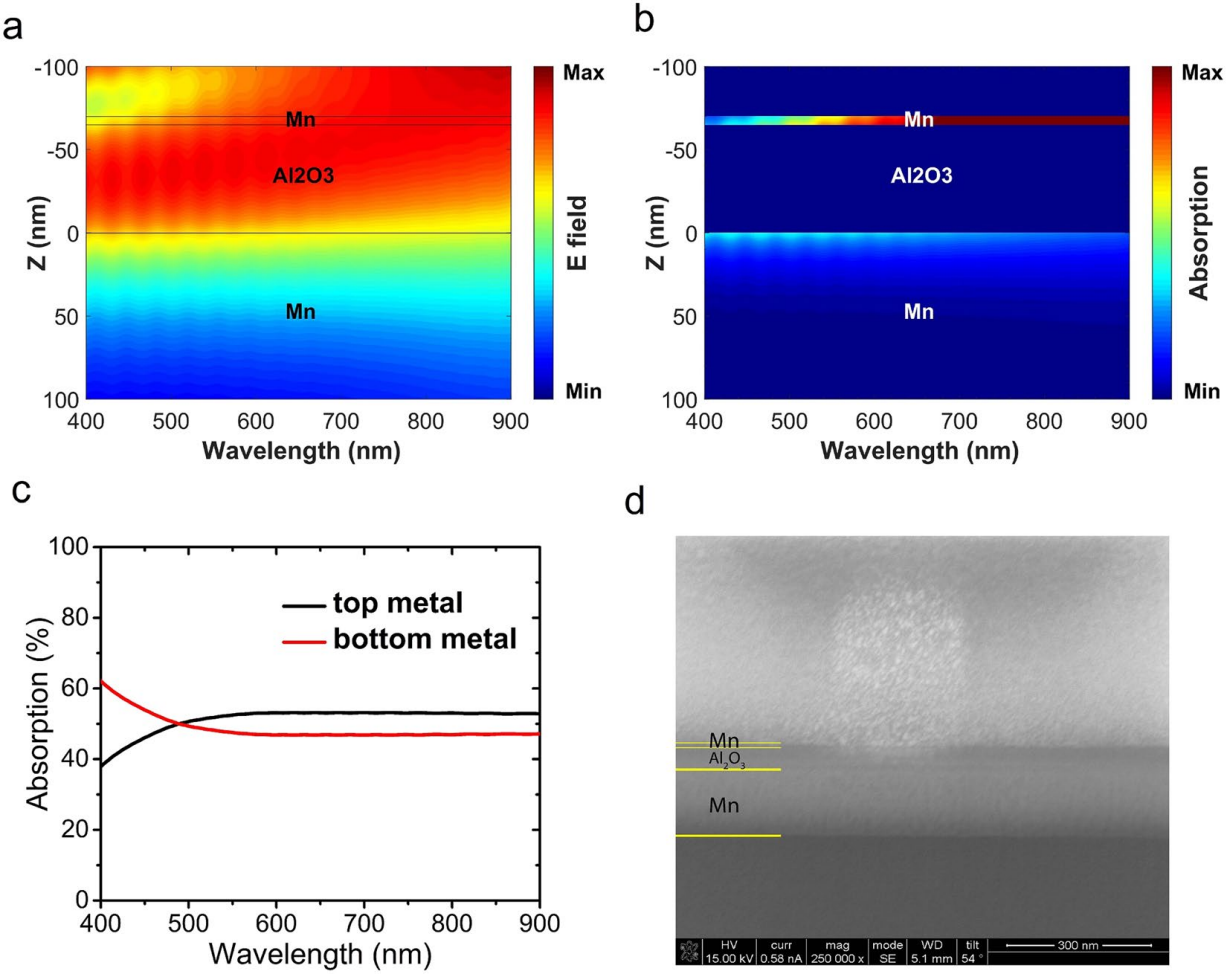
Simulated (**a**) electric field intensity and (**b**) absorption distribution in the cross section of the optimal MIM structure as a function of wavelength, (**c**) contribution of top and bottom metal layers in the light absorption and, (**d**) SEM image of the cross section of the fabricated MIM structure, milled by FIB.

To further elucidate the electromagnetic phenomena taking place in this structure, the electric field distribution inside the structure for the wavelength range of interest (400–900 nm) is depicted in Fig. 7(a). Since the structure is not changing in the lateral direction, we have only depicted the vertical spatial variation of the field inside the structure. It is obvious that a cavity resonance mode is trapped inside the structure in the dielectric region and back and forth reflections from the top and bottom metallic layers lead to a standing wave inside it.

To better observe the contribution of each layer to absorption, the absorption intensity in different layers in the structure versus wavelength is shown in Fig. 7(b). It can be seen that the absorption intensity is high at the top metal, but it is accumulated in a thin layer. In the bottom metal, on the other hand, the absorption intensity is lower compared to the top metal, but it is distributed in a thicker layer. To evaluate the total contribution of each layer to absorption, Fig. 7(c) shows the percentage of total absorption in each metallic layer, versus wavelength. It can be observed that the contribution of the top and bottom metallic layers is not significantly different. As can be seen in both Fig. 7(b,c), at lower wavelengths, the bottom layer has a larger contribution to the absorption and the absorption of the top metal layer increases at longer wavelengths. Finally, the Scanning Electron Microscopy (SEM) image of the cross section of the fabricated sample, which is milled by Focused Ion Beam (FIB) is shown in Fig. 7(d).

## Conclusion

Step-by-step design and optimization of a broadband MIM absorber is presented, geometrically and in terms of materials. The important advantage of this absorber is the use of three large area layers without any need to lithography or any repetition of MI pairs, which makes this absorber as simple as possible. It has high absorption within the whole visible range and going into the NIR region. Achieving such a response with such a simple structure is very promising for mass production. We have used and introduced Mn for the first time as the metal layer in a broadband absorber. We have shown that Mn is a promising material for broadband absorption by investigating its permittivity profile. We have shown that its complex permittivity profile is very close to that of the ideal metal for achieving perfect impedance matching to that of free space in our wavelength range of interest (400–900 nm). It was shown experimentally that the structure has an omni-directional response up to angles as large as 70 degrees, for both TE and TM polarizations. This work proved that Mn has great potential in broadband absorption structures and their deriving applications.

## Methods

### Experimental

For the fabrication of the structure, first, a typical commercial square-shaped transparent Sapphire wafer with 100 mm^2^ area has been cleaned with Piranha solution, deionized water, Acetone, and Isopropanol, and then has been dried with N_2_ flow, to be used as the substrate. Then, a 200 nm Mn film has been coated on it using VAKSIS Thermal Evaporator in the chamber pressure of 3e-6 to 5e-6 Torr and with the rate of 0.5 A/s. After that, 65 nm Al_2_O_3_ has been coated using H_2_O and Al(CH_3_)_3_ as precursors at 200 °C in an ALD reactor (Cambridge Nanotech Savannah S100). Finally, 5 nm Mn has been coated on the structure as the top metal layer using the same Thermal Evaporator as for the bottom layer, at the same chamber pressure and with the rate of 0.1 A/s. For characterization, a home-made fiber optic based reflectometer setup has been used for measuring the reflection at normal incidence up to the wavelength of 850 nm. For wavelengths above 850 nm, measurement of the reflection at normal incidence has been carried out using Fourier Transform Infra-Red (FTIR, Bruker). Reflection measurements with angled incidence and for different polarizations, transmission measurements, and material characterizations for permittivity extraction have been done using J.A. Woollam Co. Inc. VASE Ellipsometer.

### Simulations

#### TMM

The calculations of the TMM method have been carried out by dividing the total electromagnetic wave inside each layer into sum of two waves each going in opposite directions, forward and backward, both of which are perpendicular to the interface of layers. Figure 1(a) depicts the 3D schematic of the structure and Fig. 1(b) shows the cross section of the structure in xz plane. The structure is infinite in x and y directions and z axis is toward top to bottom, perpendicular to the interfaces of layers. The total electric field (*E*_*i*_) and magnetic field (*Hi*) inside each layer can be written as:

5a $${E}_{i}={E}_{i}^{+}{e}^{j{\beta }_{i}z}+{E}_{i}^{-}{e}^{-j{\beta }_{i}z}$$

5b $${H}_{i}={H}_{i}^{+}{e}^{j{\beta }_{i}z}+\,{H}_{i}^{-}{e}^{-j{\beta }_{i}z}$$

where the subscript ‘*i*’ corresponds to the number of layer, which is a number from 0 to 4 (see Fig. 1(b)), from top to bottom (including free space at both top and bottom sides of the structure with numbers of 0 and 4, respectively), and ${E}_{i}^{+}$ and ${E}_{i}^{-}$ correspond to the amplitude of the waves traveling forward (+z) and backward (−z), respectively. *β*_*i*_ is the complex propagation constant inside i^th^ layer which is equal to $\omega \sqrt{{\varepsilon }_{i}{\mu }_{i}}$ with is *ω* being the angular frequency, *ε*_*i*_ being the complex permittivity and *µ*_*i*_ being the complex permeability of the layer. *µ*_*i*_ is equal to permeability of free-space in the case of the materials we have used in this work (non-magnetic materials).

In order to relate the amplitudes in layer 0 (incident wave) to layer 4 (transmitted wave), we must multiply the interface and propagation matrices with the appropriate sequence. The propagation matrix of i^th^ layer, named as ${{\rm{P}}}_{{\rm{i}}}$, is the matrix which stands for the propagation of the wave inside i^th^ layer and transforms the field amplitudes at one end of the layer into another end, by multiplying them with their corresponding propagation coefficients, and is written as below:

6 $$(\begin{array}{c}{E}_{({z}_{i})}^{+}\\ {E}_{({z}_{i})}^{-}\end{array})={{\rm{P}}}_{{\rm{i}}}(\begin{array}{c}{E}_{({z}_{i-1})}^{+}\\ {E}_{({z}_{i-1})}^{-}\end{array})$$

7 $${{\rm{P}}}_{{\rm{i}}}=(\begin{array}{cc}{e}^{j{\beta }_{i}{d}_{i}} & 0\\ 0 & {e}^{-j{\beta }_{i}{d}_{i}}\end{array})$$

where ${d}_{i}$ is the thickness of i^th^ layer and *z*_*i*_ is the position on z axis at the end of the layer, as depicted in Fig. 1(b).

To relate the field amplitudes in two sides of interface, boundary conditions should be imposed. Since the structure is symmetric in the xy plane, there is no difference between TM and TE waves in normal incidence and even one can assume the wave to be TEM. Without any loss of generality, We suppose the electric field to be in the +x direction and, therefore, using the right hand rule, considering the fact that propagation is in the +z direction, the magnetic field has to be in the +y direction. Therefore, boundary conditions are indeed tangential boundary conditions that convey the continuity of electric and magnetic fields as follows:

8a $$({E}_{i}^{+}+{{E}_{i}^{-})|}_{z={z}_{i}}={({E}_{i+1}^{+}+{E}_{i+1}^{-})|}_{z={z}_{i}}$$

8b $$({{H}_{i}^{+}+{H}_{i}^{-})|}_{z={z}_{i}}={({H}_{i+1}^{+}+{H}_{i+1}^{-})|}_{z={z}_{i}}$$

By substituting *H* using the relation $H\,=\,\frac{n}{{Z}_{0}}E$ that holds between the electric and magnetic field, in which *Z*_0_ is the impedance of free-space, and using the right hand rule, and after doing a few simplifications, equation (8b) evolves into the following equation:

9 $$({n}_{i}\,{E}_{i}^{+}-{n}_{i}\,{{E}_{i}^{-})|}_{z={z}_{i}}=({n}_{i+1}\,{{E}_{i+1}^{+}-{n}_{i+1}{E}_{i+1}^{-})|}_{z={z}_{i}}$$

Now, by having two equations of (8a) and (9) between electric fields in both sides of interface, we can write interface matrix named as I_*i,i*+1_ which relates fields in two sides of the interface of i^th^ and (i + 1)^th^ layers, as follows:

10 $${{\rm{I}}}_{i,i+1}=(\begin{array}{cc}\frac{{n}_{i+1}+{n}_{i}}{2{n}_{i+1}} & \frac{{n}_{i+1}-{n}_{i}}{2{n}_{i+1}}\\ \frac{{n}_{i+1}-{n}_{i}}{2{n}_{i+1}} & \frac{{n}_{i+1}+{n}_{i}}{2{n}_{i+1}}\end{array})$$

Where

11 $${(\begin{array}{c}{E}_{i+1}^{+}\\ {E}_{i+1}^{-}\end{array})|}_{z={z}_{i}}={{\rm{I}}}_{i,i+1}{(\begin{array}{c}{E}_{i}^{+}\\ {E}_{i}^{-}\end{array})|}_{z={z}_{i}}$$

Therefore, the relation between the field amplitudes of the 0^th^ layer and 4^th^ layer is in the form of

12 $$(\begin{array}{c}{E}_{4}^{+}\\ {E}_{4}^{-}\end{array})=({{\rm{I}}}_{3,4})({{\rm{P}}}_{{\rm{3}}})({{\rm{I}}}_{2,3})({{\rm{P}}}_{{\rm{2}}})({{\rm{I}}}_{1,2})({{\rm{P}}}_{{\rm{1}}})({{\rm{I}}}_{0,1})(\begin{array}{c}{E}_{0}^{+}\\ {E}_{0}^{-}\end{array})$$

Then, we take ${E}_{0}^{+}$ equal to 1 as the amplitude of the incident wave, and set ${E}_{4}^{-}$ to zero, since there is no backward propagating wave in free-space at the bottom of the structure, because of no reflecting interface after it. Therefore, there will be 2 knowns (${E}_{0}^{+}$ and ${E}_{4}^{-}$) and two unknowns (${E}_{0}^{-}$ and ${E}_{4}^{+}$) that are related to each other by two equations generated from a 2 by 2 matrix.

Now, T and R of the MIM structure can be calculated as follows:

13 $${\rm{R}}={|\frac{{E}_{0}^{-}}{{E}_{0}^{+}}|}^{2},\,{\rm{T}}={|\frac{{E}_{4}^{+}}{{E}_{0}^{+}}|}^{2}$$

#### FDTD

FDTD simulations are carried out by employing Lumerical FDTD Solutions which is a commercial FDTD software package^59^. The simulations are done in a 2D simulation environment in the xy plane and a unit cell with 200 nm length in the x-direction is employed to define the structure. Boundary conditions are set to periodic in the x axis boundaries and y axis boundaries are defined as Perfectly Matched Layer (PML). The structure is excited by a plane wave in the –y direction and a linear power monitor behind the source records the data of R. There is another linear power monitor on the other side of the structure that records T.
